# Supplementary material for: Plocabulin, a Novel Tubulin Inhibitor, Has Potent Antitumour Activity in Patient-Derived Xenograft Models of Soft Tissue Sarcoma
Source: Int J Mol Sci. 2022 Jul 5;23(13):7454. doi: 10.3390/ijms23137454 (PMC9267286; doi:10.3390/ijms23137454)
Supplement: Supplementary file 1 [file ijms-23-07454-s001.zip › ijms-1773932-supplementary.pdf]

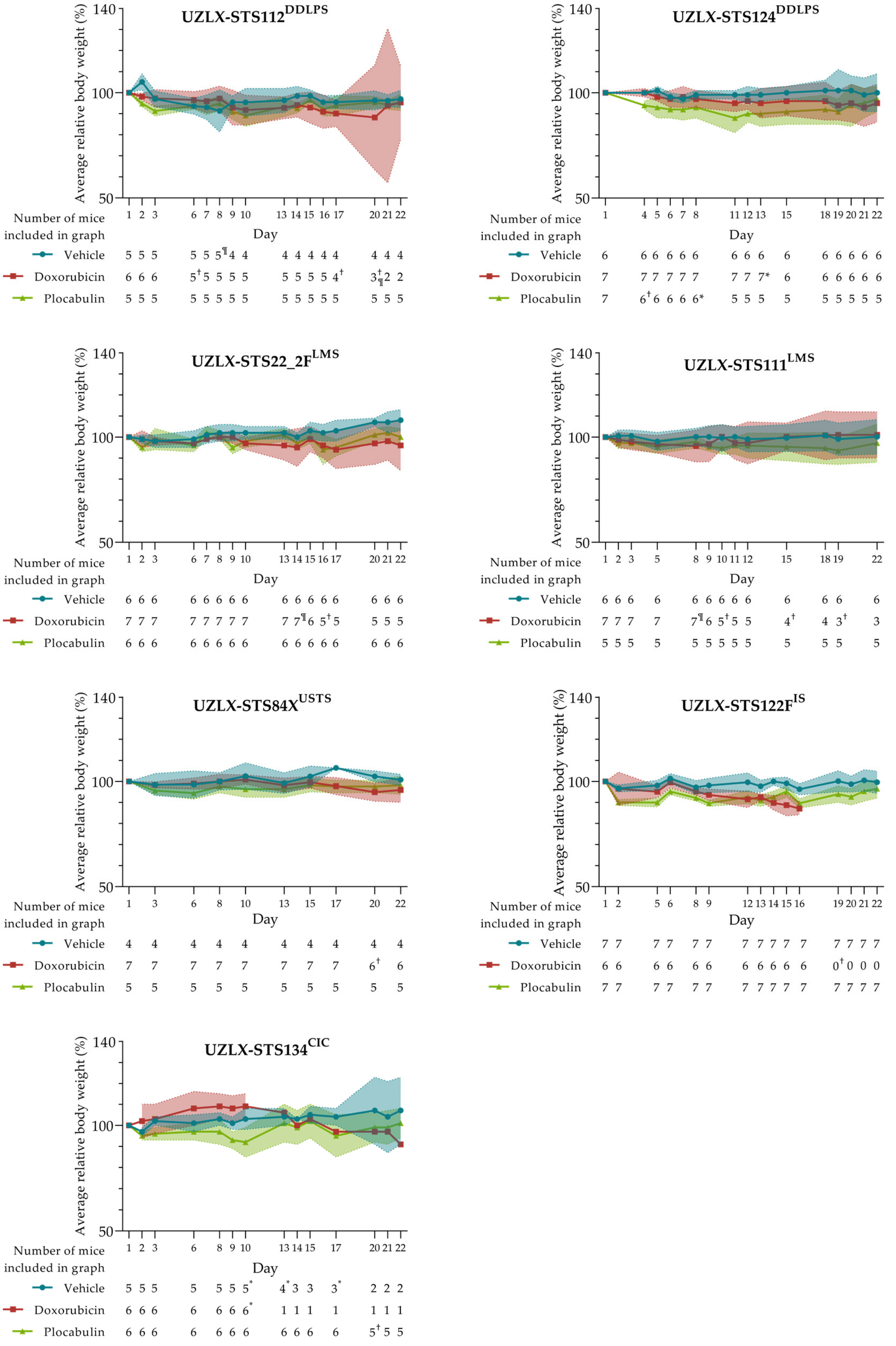

Figure S1. No severe weight loss was observed with plocabulin. Detailed overview of body weight evolution. per treatment group per model. A detailed overview of the number of mice included on each day is shown below each graph. Data graphed as mean and with 95% confidence interval as shaded area. †: mouse/mice found dead; * mouse/mice sacrificed due to

tumour volume exceeding ethical limits; ¶: mouse/mice sacrificed due to illness.
